# The evolution of nanomedicine: The rise of next-generation nanomaterials in cancer nanomedicine

**DOI:** 10.1126/sciadv.adx1576

**Published:** 2025-10-22

**Authors:** Helen Forgham, Yixin Chang, Yao Wang, Jiayuan Zhu, Liwei Liu, Heather Biggs, Aleksandr Kakinen, Yuhao Jiang, Xinru You, Kristofer J. Thurecht, Shaohua Ma, Lining Arnold Ju, Wei Tao, Thomas P. Davis, Joyce Y. Wong, Ruirui Qiao

**Affiliations:** ^1^ Australian Institute of Bioengineering & Nanotechnology, The University of Queensland, Brisbane, Queensland 4072, Australia.; ^2^ School of Biomedical Engineering, Faculty of Engineering, The University of Sydney, Darlington, NSW, 2008, Australia.; ^3^ Center for Nanomedicine and Department of Anaesthesiology, Brigham and Women’s Hospital, Harvard Medical School, Boston, MA, USA.; ^4^ Centre for Advanced Imaging (CAI), ARC Training Centre for Innovation in Biomedical Imaging Technology, The University of Queensland, St. Lucia, Queensland 4072, Australia.; ^5^ Institute of Biopharmaceutical and Health Engineering (iBHE), Tsinghua Shenzhen International Graduate School (SIGS), Tsinghua University, Shenzhen 518055, China.; ^6^ Division of Materials Science and Engineering, Boston University, Boston, MA, USA.; ^7^ Department of Biomedical Engineering, Boston University, Boston, MA, USA.

## Abstract

The nanomedicine field continues to gain momentum, with several groundbreaking clinical trials underway. However, despite the promise of advanced antifouling nanoparticles incorporating poly(ethylene glycol)—a key component in the development of COVID-19 vaccines—the clinical translation of nanomedicine remains limited. This is primarily due to the relatively low delivery efficacy, with passive targeting relying on the enhanced permeability and retention effect, and active targeting leading to only modest improvements in target tissue accumulation. Improving the targeting, biocompatibility, and functionality of nanoparticles has the potential to create more effective, personalized, and minimally invasive therapies. This review aims to highlight the rise of a previously unidentified order of immune-minded nanomaterials and explores how mechanobiological principles and biomechanical nanotools are revolutionizing our understanding of nano-bio interactions in relation to disease. By considering mechanical properties such as stiffness, surface topology, and behavior under physiological flow conditions, researchers can better engineer nanoparticles for improved therapeutic outcomes.

## Teaser

Cancer nanomedicine is evolving, as researchers harness mechanobiology to improve targeting, delivery, and therapeutic response.

## INTRODUCTION

**Fig. 1.**
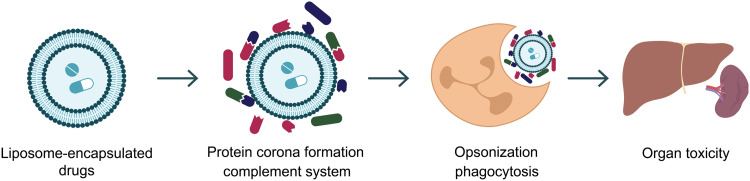
First NP formulations developed. The first generation of NPs was lipid formulations, which were prone to corona formation and rapid clearance by the liver and kidneys, promoting toxic accumulation in these organs.

Nanomedicine represents the pinnacle of modern medicine, blending physical, chemical, and biological features seamlessly in the creation of tiny structures [nanoparticles (NPs)] with medical delivery potential (Fig. 1).

**Fig. 2.**
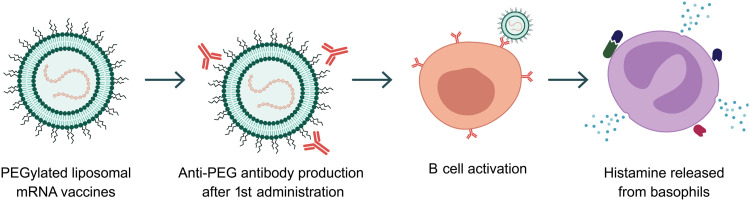
Inclusion of PEG to NP formulations. The inclusion of PEG provided better stability and improved both circulatory times and cellular uptake. PEGylated liposomal mRNA vaccines used against COVID-19 saving countless lives. However, immunogenic problems with PEG exist through production on anti-PEG antibodies, B cell activation, and release of histamine by basophils.

The 1960s brought the first wave of NPs in the form of liposomes (Fig. 1). Conceptualized by Bangham *et al.*, these first-of-their-kind transformative NPs were proof of concept that NPs could be used to enhance efficacy and reduce off-target toxicity. However, what Bangham cultivated was the rationale for prolific pursuit of the “magic biological delivery bullet” (*1*). It was not until the 1980s that NPs really began to demonstrate their potential in cancer treatment (*2*). Coincidentally, during this time came the notion of a cancer-fueled type of structural anarchy that NPs could exploit for better therapeutic outcome. Thus was born the concept of the “enhanced permeability and retention effect (EPR),” still classed as the most recognized route of tumor uptake by clinically approved formulations (*3*). 1995 saw the first Food and Drug Administration (FDA)–approved flagship NP for cancer. Doxil, a liposomal formulation encapsulating doxorubicin, remains in the clinic to this day and has helped many patients across the cancer landscape; however, Doxil is not the magic biological delivery bullet first imagined. During the late 1970s, poly(ethylene glycol) (PEG) first showed promise when conjugated to proteins, demonstrating increased water solubility and reduced kidney clearance (*4*). Thus, when materials that could help NPs to evade capture became the focus in the late 90s, PEG-functionalized NPs (Fig. 2) were forefront in this previously unknown era of “stealth.” Inclusion of PEG broadened the field of NPs and their capacity not only as therapeutic delivery vehicles but also as diagnostic probes and multipurpose vehicles capable of simultaneously carrying therapeutics and diagnostic entities.

At the turn of the millennium, under the presidency of Bill Clinton, realization of the groundbreaking possibilities arising from nanotechnology in multiple spheres including medicine captured national and global attention. At risk of notable advances being fragmented and limited in impact, US government leaders, scientists, and industry stakeholders set about forging the administration of a comprehensive, strategic approach to foster research, development, and the commercialization of nanotechnology. The result: launch of the National Nanotechnology Initiative (in 2000) (*5*)—a coordinated national framework designed to harness the full potential of nanotechnology. Many powerful countries followed suit—China, Japan, and those of the European Union committing substantial investment into nanotechnology, optimizing design, demonstrating clinical capabilities, and offering a diverse range of previously unidentified polymer materials and methodologies aimed at providing various medical solutions.

As research continued into 2010, prominent groups began to appear producing innovative study data, demonstrating the importance of simple production methods using biodegradable materials and showcasing how physicochemical properties such as size, shape, and surface charge greatly influenced biodistribution and bioavailability in vivo. Researchers increasingly recognized that a deep understanding of these characteristics was essential for the effective clinical translation of nanomaterials. Ahead of the curve at the time were people such as O. Eniola-Adefeso, who published a seminal review on the importance of size (*6*) and led her field in identifying the most effective size of spherical NPs aimed at targeting vasculature walls. Her group was among the first to identify that spherical NPs measuring 2 to 5 μm were optimal for targeting medium to large arteries, particularly in the context of cardiovascular diseases (*7*). She was also pivotal in the development of targeted spherical NPs to treat atherosclerosis in arteries in areas with high disturbance in blood flow (*7*). Here, spheres with a diameter of 2 μm seem to be optimal for effectively margination to the vessel wall in areas of disturbed bulk blood flow.

Meanwhile, fellow trailblazer, S. Mitragotri and his group focused on the impact of shape. His group demonstrated that rod-shaped NPs had enhanced specific uptake and reduced nonspecific uptake across various breast cancer cell lines (BT-474, SK-BR-3, and MDA-MB-231) compared to spherical counterparts (*8*). They we also one of the first to demonstrate how shape influenced binding strength to epithelium, demonstrating that rods exhibited greater binding strength and selectivity (maximizing targeted delivery) to brain and lung endothelium compared to spherical NPs (*9*). Around the same time it was determined that larger contact areas, such as in elongated shapes, reduce inhalation effectiveness for lung targeting—due to increased van der Waals forces, which promote particle aggregation and clumping, thus showing the subtle nuances that exist when considering different delivery angles (*10*).

While Felgner *et al.* (*11*) in 1987 were one of the first to report on a positive charge being favorable in uptake and facilitating the expression of the DNA, later additional studies found excessive charge to be toxic and highly immunogenic. Research in 2010 was therefore more tailored toward how charge affected the interactions with immune cells and the mononuclear phagocyte system and what was the optimal charge for the desired therapeutic outcome. Another influential figure during this period was T. Davis, a pioneer of reversible addition–fragmentation chain transfer polymerization. His group made noteworthy contributions to the design of polymer-based NPs, investigating the effects of shape, charge, and surface properties (*12*). They also demonstrated how polymer coatings could stabilize inorganic NPs and be used to fine-tune surface charge through coassembly of charged and neutral polymers (*13*). One of the most prestigious papers of the time was a review piece published in The New England Journal of Medicine by nanoparticle sovereignty, W. Chan and his group (*14*). This review provided a comprehensive appraisal of the various nanomaterials under development—with discussion of tunable optic, electronic, magnetic, and biological properties required in the development of in vivo therapeutic delivery or in vitro diagnostics.

Before 2019, the inclusion of PEG in nanoformulations the years leading up to the COVID-19 pandemic saw a cluster of first-of-their-kind nanomedicines reaching the clinic; perhaps none more so exciting than patisiran, a gene-based short interference RNA used in the treatment of hereditary transthyretin-mediated amyloidosis (*15*). Before NP development, gene therapies that could focus specifically on a single target were not biologically viable. However, once properly encapsulated by an NP carrier, the field of gene-based medicines began to flourish—just in time to save hundreds of thousands of lives. When the COVID-19 pandemic hit, mRNA gene-based vaccines were still very much under preclinical evaluation but were showing promising results against the deadly Ebola virus. However, it was COVID-19 and the scramble to find a vaccine that could stop its effect that made nanomedicine and gene therapies famous throughout the world.

Today, PEG grafting remains the most commonly used surface modification antifouling approach for clinically approved NPs and those currently undergoing clinical trials (Tables 1 and 2). Because of its ethylene glycol subunit (-CH_3_-CH_3_-O-), PEG is highly hydrophilic, with approximately three water molecules binding to each subunit. It is best characterized by its stability-enhancing attributes, low cytotoxicity, and superior antiopsonization properties that temper protein corona formation (*16*). When PEG chains were examined (5 to 20 kDa), the longer PEG chain lengths show increased grafting density due to higher coverage rates, while shorter PEG chains demonstrate longer circulatory times and improved cellular uptake (*17*). Meanwhile, high grafting densities of hyaluronan polymers encourage low protein adsorption more so than low (*18*), meaning both grafting density and long circulatory times are important design considerations and highlighting the challenges in developing the most effective PEGylated NPs for biological applications. Nevertheless, even with the current biological limitations, which include poor biodegradability, potential for accelerated blood clearance with repeated doses, histological abnormalities, and immunogenicity (*19*), PEG has maintained its status as the “gold standard” biofriendly NPs addition for the past 30 years. However, immunogenic issues could pose a demonstrable long-term problem. Currently identified issues with clinically approved PEGylated NPs include the potential for anaphylaxis through complement activation–related pseudoallergy and immunoglobulin E (IgE)–activated basophils and propensity for a patient to develop acute high levels of systemically reactive anti-PEG antibodies (*20*). The occurrence of anti-PEG antibodies, including IgM, IgG, and, to some extent, IgE classes, within the population is growing exponentially. These antibodies can form following repeated exposure to PEGylated products, such as cosmetics, food items, or vaccines, including those developed for COVID-19.

**Table 1.** Clinical trials aimed at actively seeking to initiate the immune system. SARS-CoV-2, severe acute respiratory syndrome coronavirus 2; DENV, dengue virus; CAR T cell, chimeric antigen receptor T cell.

Nanoparticle type	Payload	Approved application	Clinical trial phase	ClinicalTrials.gov identifier	References	Study start	Study completion
Lipid nanoparticle	Messenger RNA (mRNA) encoding a prefusion nonstabilized spike protein	SARS-CoV-2 vaccine	Phase I/II	NCT04566276	(*126*)	03/05/2021	06/12/2022
Lipid nanoparticle	Recombinant spike protein	SARS-CoV-2 vaccine	Phase III	NCT04583995	(*127*)	28/09/2020	29/03/2022
				NCT04611802		27/12/2020	15/12/2023
Self-assembling recombinant protein nanoparticle adjuvanted with AS03 or aluminum hydroxide	Immunogens	SARS-CoV-2 vaccine	Phase I/II	NCT04750343	(*128*)	03/02/2021	06/2022 (estimated)
				NCT04742738		20/01/2021	07/07/2022
Gold nanoparticle	T cell priming specific cocktail of coronavirus peptides	SARS-CoV-2 vaccine	Phase I	NCT05113862	(*129*)	10/01/2022	15/09/2022
Gold nanoparticle	T cell priming specific cocktail of dengue virus peptides representing all 4 DENV serotypes	Dengue virus	Phase I	NCT04935801	(*129*)	02/08/2021	15/09/2022
Poly(lactic-co-glycolic acid) (PLGA) nanoparticle	New York esophageal squamous cell carcinoma-1 (NY-ESO-1) cancer-testis antigen peptides	NY-ESO-1–positive cancers	Phase I	NCT04751786	(*130*)	11/01/2021	07/2025 (estimated)
RNA-lipid complex nanoparticle	Uridine nucleoside mRNA encoding the target antigen recognized by the CAR T cells	Relapsed or refractory solid tumors	Phase I	NCT04503278	(*131*)	16/09/2020	01/2040 (estimated)

**Table 2.** Clinical trials aimed at evading recognition by the immune system.

Nanoparticle type	Payload	Approved application	Clinical trial phase	ClinicalTrials.gov identifier	References	Study start	Study completion
Poly(ethylene glycol)-polyethyleneimine cholesterol lipopolymer	DNA plasmid encoding IL12 gene	Epithelial ovarian cancer	Phase I/II	NCT03393884	(*132*)	05/09/2018	30/11/2025 (estimated)
PEGylated liposomes	Coagulation factor VIII	Hemophilia A	Phase II	NCT04592692	(*133*)	23/12/2019	31/5/2022 (estimated)
Exosome	Extracellular vesicles from mesenchymal stem cells	Coronavirus infection	Early phase I	NCT05787288	(*134*)	23/01/2023	23/01/2025 (estimated)
Exosome	Allogenic adipose mesenchymal stem cells derived extracellular vesicles	Novel coronavirus pneumonia	Phase I	NCT04276987	(*135*)	15/02/2020	31/07/2020

While Omata *et al.* (*21*) suggest that the antigen-specific immune response of mRNA–lipid NP (LNP) vaccination is not notably affected by anti-PEG antibodies, Liu *et al.* (*22*) recently determined that preexisting anti-PEG antibodies reduce vaccine effectiveness, affect pharmacokinetics, and increase complement activation, underscoring the importance of identifying preexisting anti-PEG antibodies to enhance vaccine performance, ensuring patient safety, and guiding the development of improved therapeutic approaches. In general, these concerns have sparked much debate regarding the future of PEGylation, with some questioning whether the technology may become outdated, especially as anti-PEG antibodies become more widespread. In attempts to improve PEG, modern designs have arisen including branched PEG, which can resist component of complement 3 (C3) protein absorption and improve coverage with higher-density moieties, avoiding complement activation (*23*), as well as countering accelerated blood clearance upon accumulated administrations (*24*). Meanwhile, polyoxazoline, the product of cationic ring-opening polymerization of 2-oxazoline monomers has also received growing interest due to excellent water solubility, biocompatibility, and in vivo stealth properties (*25*).

Often, NPs fail in early clinical trials due to poor consideration of innate immunity, including rapid clearance by monocytes and neutrophils, and complement activation (*26*). At the heart of the problem is an inability to control fouling and inadequate understanding of material properties under in vivo conditions. This Review summarizes the mechanisms of NP delivery into tumors, focusing on engineering strategies and recent studies over the last 5 years aimed at moderating fouling and regulating immune responses to advance next-generation nanomedicines. We also highlight the emergence of next-generation nanomedicines that leverage mechanobiology and its capacity to unearth previously unidentified, targetable cellular mechanisms and guide the design of immune-savvy NPs beyond the EPR effect, as well as use advanced mechanobiology techniques to deepen our understanding of nano-bio interactions within the bloodstream and the tumor microenvironment.

### Mechanisms of NP delivery

The mechanisms underpinning vivo NP delivery involve passive and active targeting (Fig. 2). Passive targeting is reliant on the EPR effect. The EPR effect is a biological paradigm driven by the idea of abnormal vascularization resulting from rapidly growing solid tumors that exhibit structurally disorganized, highly permeable defective blood vessels (*27*). This together with the compression of lymphatic vessels by proliferating tumor cells is considered to impair lymphatic drainage, limiting internal NP clearance (*28*). Collectively, these factors are thought to lead to prolonged NP-therapeutic exposure within the tumor tissue, and hence, the EPR effect has been the focus of NP design and clinical translation over the past several decades (*29*). Nanoparticles less than 100 nm are regarded as optimal for successful passage through the interendothelial gaps in the leaky vascular; however, the clinical success of EPR-based nanomedicines remains modest, with a seminal investigation of 232 data sets by Wilhelm *et al.* (*30*), demonstrating that an average of less than 1% of injected NPs reaches tumors. Nevertheless, this average was recorded in all honesty for outcomes, which were excessive in contrast, given that studies that fell below 1% were mixed with others that achieved an uptake more than and above 10%. While even 10% may appear somewhat low, small-molecule drugs and antibodies typically accumulate in tumors at rates of only 1 to 10% of the injected dose, indicating that that this is an issue requiring attention and further optimization in all therapeutic sectors. Notwithstanding the results of the Wilhelm *et al.* findings, the consensus was that the extent of NP accumulation varied considerably based on factors such as NP size, charge, and composition, as well as the specific tumor model and experimental conditions used—elements with the definitive capacity for greater enhancement moving forward (*30*).

Recently, Nguyen *et al.* (*31*) reported that lymphatic drainage in tumors is not dysfunctional as previously suggested. Specifically, NPs larger than 30 nm predominantly exit through intratumoral lymphatics, whereas smaller NPs are cleared through peritumoral lymphatics (*31*). In attempts to improve delivery, mechanisms that actively facilitate endothelial transcytosis have been explored [active transport and retention (ATR)]. The ATR approach is based on the principle that rather than NPs reaching the tumor tissue through interendothelial gaps, tumor uptake is a result of endothelial transcytosis, vesiculo-vacuolar organelles and/or immune cell migration. Moreover, NPs are held through collective interactions with tumor-associated macrophages (TAMs), which can act as NP reservoirs (*32*), and through cellular and extracellular matrix conditions that trap the NPs within the tumor microenvironment for longer retention (*33*). However, the ATR approach also has its challenges, given that the characteristics of both the tumor and the NPs are considered highly influential to success (*34*).

**Fig. 3.**
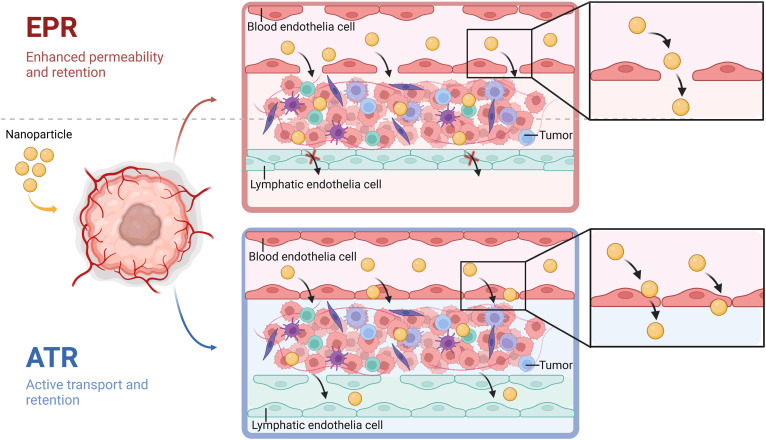
Mechanisms of NP delivery into tumors. The EPR effect states that NPs gain access to the tumor site through interendothelial gaps and are held within the tumor due to poor lymphatic drainage. The ATR concept promotes the idea that NPs use active endothelial transport to pass into the tumors and exit through lymphatics.

All things considered, the current consensus seems to suggest that whether the EPR or ATR (Fig. 3) is the best approach depends on tumor architecture. This is perhaps best exemplified by the work of Zhu *et al.* (*35*), who examined the tumor accumulation of ferritin NPs in 32 mouse tumor models (brain, breast, pancreas, liver, and skin, respectively). The study identified that 70% of the tumor models had low NP accumulation and 30% high accumulation. While the 70% group was shown to rely on the ATR effect for uptake due to absence of notable EPR effect, the 30% group appeared to favor the EPR over the ATR. As we begin to understand the subtle biological differences in tumor development and growth, there is an urgent need to develop a generation of NPs to match these previously unidentified challenges in delivery: NPs with better surface design that encourage enhanced penetration—taking advantage of mechanobiology mechanisms.

### Emergent nanomedicine

**Fig. 4.**
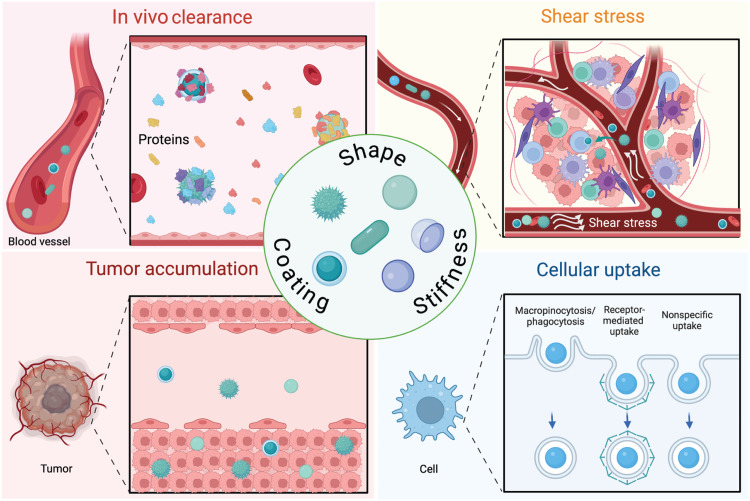
Key biological areas where optimization of NP shape, stiffness, and surface coating can enhance performance. These design parameters can be strategically tuned to improve cellular uptake, biodistribution, circulation time, immune evasion, and targeting efficiency.

**Fig. 5.**
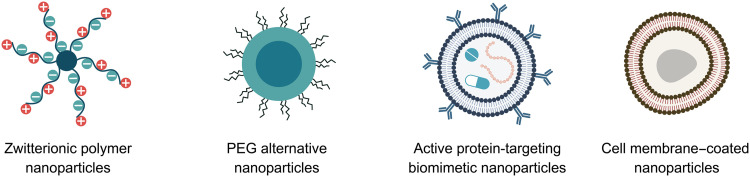
Emergence of alternative NPs under design. The propensity for production of anti-PEG antibodies continued issues with immune clearance and/or potentially life-threatening allergic reactions has provided the impetus to seek alternative NP design approaches.

Driven by breakthroughs in biological understanding of the gross heterogenicity of tumor tissue and the tumor microenvironment, as well as cutting-edge innovations in materials science and biomechanical tool kits, the field of nanomedicine has grown exponentially since the early days of LNPs, with design more attuned to the problematic areas associated with inadequate therapeutic potential (Fig. 4). In this section, we discuss emerging approaches that aim to better navigate the immune system and provide superior targeting toward tumor tissues (Fig. 5).

#### Nonionic PEG alternatives

To overcome the current shortfalls of PEG, we explored other nonionic branched polymer alternatives including poly(acrylate)s (*36*) and poly(oligo(ethylene glycol)methyl ether methacrylate) (POEGMA) (*37*). POEGMA is developed through substitution of long PEG for short oligoethylene glycol oligomers. POEGMA is less antigenic than PEG and has demonstrated excellent gene silencing in vitro and in vivo with negligible immune responses (*38*–*40*). Furthermore, it has been shown that reducing the number of repeating units to three in POEGMA-protein conjugates lowers reactivity, as does inclusion of redox-responsive cleavable disulfide bonds (*23*, *41*). In addition, polyglycerol (PG), a small, single molecule with three carbons and two hydroxyl groups that facilitate branching has shown promising results for immune evasion (*42*). PG displays a different linear, hyperbranched arrangement to hyperbranched PEG derivatives, which was demonstrated to facilitate successful immune evasion, attributed to prevention of both protein corona formation and macrophage uptake—a result that is independent of NP size and markedly better than PEG. Fluorinated polymers are developed from perfluoropolyether building blocks. They are highly hydrophobic, which helps encourage stability in serum and endows them with excellent repellent properties—a study recently demonstrating that fluorinated polymer coating on gold NPs reduced adsorption of immune reactive proteins and phagocytic uptake (*43*). Meanwhile, hydrophilic sulfoxide-containing polymer—poly(2-(methylsulfinyl)ethyl acrylate)—has demonstrated a reduction in both protein corona buildup and macrophage-related phagocytosis, as well as a 2.5-fold increase in circulatory time (*44*, *45*).

#### Zwitterionic NPs

Zwitterionic polymers (ZPs) (Fig. 1C) have received considerable attention for their ability to evade immune system detection, provide effective antifouling, and lower circulatory clearance rates. In contrast to PEG, which gains its antifouling properties from a hydration layer created by hydrogen bonding, ZPs achieve their antifouling effect through a hydration layer formed by electrostatic interactions. This is due to the evenly distributed opposing charge groups along the polymer backbone’s repeating units (*46*). Polyzwitterions (PZPs) contain zwitterionic moieties as monomers (*47*). Each side chain features a positively charged motif, typically an amino group or quaternary ammonium group for betaines, and a negatively charged motif such as a carboxylate, phosphoryl, or sulfonate group. The arrangement ensures an overall neutral charge, rendering the structure highly polar. This together with hydration layer forming electrostatic interactions between PZPs and water molecules is said to prevent fouling (*48*). However, the degree of hydrophobicity in PZPs is correlated to the distance between the two charged moieties—a shorter distance resulting in a denser hydration layer (*49*). Polycarboxybetaine (PCB) is a type of PZP. Surfaces coated with PCB have demonstrated the ability to resist nonspecific protein adsorption in 100% human plasma, serum, and whole blood (*50*). However, this comes with a caveat in that the length of the carbon spacer between the ammonium and carboxylate groups influences their nonfouling characteristics. PCBs with a spacer length of one or two show excellent nonfouling properties, whereas those with a spacer length of three or more loses fouling effectiveness (*51*).

In a study by Li *et al.* (*52*) aimed at improved lymphatic delivery, PCB-modified liposomes were compared against PEGylated liposomes encapsulated with highly immunogenic enzyme l-asparaginase (ASP). Both PCB and PEG were conjugated using the same heterobifunctional cross-linker (*N*-α-maleimidoacet-oxysuccinimide ester), ensuring uniform linkage chemistry and comparable NP sizes. PCB-ASP markedly reduced antiprotein IgG levels and entirely prevented the generation of anticonjugate IgG, a notable improvement over PEG-ASP. These findings support not only the superior lymphatic transport and bioavailability of PCB-modified liposomes but also their potential for inducing immunological tolerance.

In a more recent landmark study by Luozhong *et al.* (*53*), PCB was conjugated to LNPs (PCB-LNPs) as an alternative to PEG lipids for mRNA delivery, aiming to improve transfection efficiency and reduce immunogenicity. PCB-LNPs outperformed PEG-LNPs both in vitro and in vivo. In primary human T cells isolated from peripheral blood mononuclear cells, mRNA encoding tdTomato yielded stronger fluorescent expression when delivered via PCB-LNPs compared to PEG-LNPs. In vivo, repeated systemic administrations in Ai14 mice demonstrated that PCB-LNPs are capable of more potent Cre recombination—as determined through ex vivo analysis of extracted splenic cells, even after multiple doses. Moreover, PCB-LNPs were able to show effective reduction in the accelerated blood clearance effect—a known phenomenon of PEG-LNPs upon second administration in patients (*54*).

Meanwhile, an innovative type of block copolymer, poly(sulfobetaine methacrylate)-block-poly(ɛ-caprolactone) (PSB-PCL), has been used to produce micelles with systematically varying alkyl substituent aimed at optimizing the balance between protein resistance and cell membrane affinity. PSB4-PCL micelles, characterized by a moderately extended chain length, exhibited affinity for cell membranes while resisting protein fouling. This combination resulted in prolonged circulation, swift cellular uptake, efficient transcytosis, increased tumor accumulation, and deep penetration within the tumor tissue. Furthermore, when loaded with paclitaxel (PCL), PSB4-PCL micelles demonstrated improved potency in a subcutaneous triple-negative breast cancer model with systemic toxicity (*55*).

Biomimetics refers to the synthesis of materials that mimic those occurring in nature (Fig. 1C) (*56*). ZP, inspired by natural biological components, is emerging as a promising replacement for PEG as demonstrated with phosphatidylserine, a naturally occurring lipid-containing immune-responsive phosphoserine (PS) head group capable of initiating immune tolerance (*57*). In nature, during apoptosis, PS typically resides on the inner surface of the plasma membrane and translocates to the outer membrane, which functions as an engulfment signal for phagocytic cells. However, through phagocytosis, PS signals to phagocytic cells, orchestrating immune tolerance toward future encounters with PS phospholipids. Taking advantage of this phenomenon, zwitterionic PS-mimetic polymers have been developed that can effectively combine zero fouling with immunomodulatory properties, reducing phagocytic clearance and delivering an immunogenic payload (*58*). Recent improvements in mRNA delivery to secondary lymphoid organs have also been reported using this tactic. Specifically, LNP formulations modified to include alcohol-soluble PS molecules with various fatty acid chains (*59*), or 1,2-dioleoyl-*sn*-glycero-3-phospho-l-serine, resulting in selective high transfection within the spleen (*53*).

While ZPs have huge potential for immune navigation, there unfortunately remain certain design hurdles that must be overcome if we are to see them arrive in the clinic. The main issue in this respect is the complex synthesis methods, which affect reproducibility, lead to batch-to-batch variation, and limit the potential for up scalability.

#### Cell membranes

Although advancements in bottom-up nanoengineering and surface chemistry have been apparent, reductionist functionalization strategies still fall short of mimicking the complex biological interfaces NPs must navigate within the blood and tissues. To counter this, an alternative biomimetics approach includes using membranes from various cell types as camouflage for NPs. To extract the membrane, well-established ultrasound, homogenization, freeze-thaw methods, or hypotonic agents are used. For fusing cell membranes with NPs, four common techniques are applied, including coextrusion, sonication, microfluidic electroporation, and electrostatic attraction. Among these, coextrusion is widely used for producing membrane-coated NPs by passing them through an extruder equipped with porous polycarbonate membranes. This process ensures efficient fusion of the membranes and NPs.

#### Red blood cells

Red blood cells (RBCs) are oxygen transporters with longevity of ~120 days. The cell structure facilitates flexibility, enabling navigation through narrow capillaries and efficient organ clearance, such as the liver and spleen (*60*). NPs designed to imitate the structural and functional characteristics of RBCs have shown promise in structural plasticity and prolonging circulatory times (*61*). RBCs are recognized as self through various cell markers that shield them from the innate immune system. Cloaking approaches have demonstrated positive results for theranostic NP approaches using synthetic polyester, poly(lactic-co-glycolic acid) (PLGA) carrying NPs tirapazamine and curcumin, combining chemotherapy and hypoxia methods (*62*), and PCL-PEG-PCL triblock copolymer coloaded with IR780 near-infrared dye and docetaxel for imaging-guided cancer photo-chemo combination therapy (*63*). In a melanoma study aimed toward photodynamic-immunotherapy targeting of tumors, protein phosphatase Pph3 (P2-PPh3), a positively charged conjugated polyelectrolyte with dual functions (fluorescence and photosensitizer) was combined with negatively charged, Toll-like receptor 3 agonist polyinosinic acid to form NPs through electrostatic interaction. PLGA was introduced into the complex by ultrasonication to provide further stability, and the RBC coat was applied (*64*). In mouse studies, the number of M1 macrophages at the tumor site was markedly increased by the RBC coat, particularly in the light-treated group, giving credibility to the suggestion that the NPs were able to promote differentiation to a proinflammatory phenotype. NPs endowed with an RBC coating can also be further modified with targeting ligands. In a cervical cancer photo-chemo combination therapy study, graphene oxide loaded with a photosensitizer (indocyanine green) and the chemotherapeutic drug, doxorubicin as internal NPs, were encapsulated within an RBC membrane shell functionalized with folic acid—for selective targeting of tumor cells through a lipid-insertion strategy (*65*). Functionalization with targeting ligands can also help cross physiological barriers such as the blood-brain barrier, delivering drugs to treat brain cancer. This was recently done using a 1,2-distearoyl-sn-glycero-3-phosphorylethanolamine- polyethylene glycol with an average molecular weight of 2000 Daltons and functionalized with Maleimide (DSPE-PEG_2000_-Mal ) nanoformulation with conjugated cysteine residues of T7 bacteriophage and asparagine, glycine and arginine (Asn-Gly-Arg )and anti-epithelial cell adhesion molecule (EpCAM) functionalized RBC-coated PLGA NPs delivering doxorubicin (*66*). Collectively, these studies demonstrate RBC coatings as a promising immune evasion strategy, and their abundance in blood ensure that they are a sustainable source for upscaling future studies. However, several concerns must be addressed before this technology can be translated into clinical applications. Rigorous quality control during RBC collection and processing remains complex, particularly regarding potential contamination. In addition, factors such as RBC alloantibodies, hyperhaemolysis syndrome, ABO antigens, and Rhesus (Rh D) antigens must be carefully considered before advancement into clinical trials to avoid potential life-threatening transfusion-based reactions.

#### Platelets

As the product of megakaryocytes in the bone marrow, platelets display a disk-shaped morphology with a diameter of ~2 to 4 μm (*67*). Platelet membrane coatings are increasingly adopted in various NP-led therapies, diagnostics, and dual-purpose theranostics that target cancer. Platelet membranes coated onto docetaxel-loaded PLGA NPs have been used to improve drug release profiles and targeted accumulation in lung cancer (*68*). Studies attempting combination chemo-photothermal therapy for breast and liver cancer have also demonstrated compelling results for doxorubicin encapsulated in platelet-coated polypyrrole and PLGA NPs (*69*, *70*). NPs’ membrane coatings can also comprise a combination of RBCs and platelets. This method was applied to gold NPs developed via the seed-mediated growth method (*71*). The gold NPs were functionalized with a heterobifunctional polymer made of PEG with two functional end groups—an amine group and a thiol group before placement of an RBC or platelet-only coat or a combined platelet RBC coat. NPs containing platelet-only coats were more likely to be phagocytosed when compared to RBC-only and platelet RBC–combined coatings, suggesting that RBC membranes were more effective in orchestrating immune evasion (*71*). These study results contrast with another where platelet membrane–coated zinc nitrate hexahydrate/2-methylimidazole metal-organic frameworks (MOFs) carrying small interfering RNA (MOF-siRNA) reduced phagocytosis by J774 macrophages to the same degree as RBC-coated MOF-siRNA (*72*). Platelet membrane coatings were shown to attenuate both interleukin-6 (IL-6) and tumor necrosis factor–α (TNFα) considerably more than noncoated MOF-siRNA and marginally more than RBC-coated MOF-siRNA. Looking at these studies side by side highlights the variability that can be observed between different NP compositions and tumor models. Moving forward, it is therefore important that we gain full insight into the complex influence of platelets on tumor progression and use this previously unknown knowledge to lead investigations of their wider potential applications in oncology.

#### Mesenchymal stem cells

Mesenchymal stem cells (MSCs) reside in bone marrow, fat tissue, and blood. They are also found in various tissues during reproduction, such as the umbilical cord, placenta, and amniotic fluid. The diverse tissue origins contribute to MSCs containing a wide range of potential receptors and cytokines compared to all other cells. Both MSCs and MSC-secreted exosomes strongly attract tumor cells and demonstrate inflammatory migratory effects, enhancing their targeting ability (*73*). Bone marrow–derived MSCs have been used as a coating for PLGA NPs delivering paclitaxel, demonstrating substantial enhancement of therapeutic efficacy (*74*). In addition, hollow gold NPs synthesized via the reduction of gold salt on the surface of cobalt NPs have been developed to carry doxorubicin (*75*). The NPs were further functionalized with glycoprotein tumor marker mucin-1 to deliver a theranostic approach against metastatic breast cancer. In vivo experiments demonstrated good tumor accumulation, and excellent Computed Tomography (CT) potential was observed up to 24 hours postadministration. Zeng *et al.* (*76*) showed that MSCs could effectively deliver SiO_2_ NPs containing photosensitizers (PS) to breast tumors for Photothermal therapy (PTT), achieving efficient tumor destruction with minimal side effects. However, the challenge remains in enhancing the penetration depth of PTT, particularly for tumors that are deeply seated or resistant to heat-induced apoptosis. The same challenge applies to other approaches, such as the use of synthetic microcapsules loaded into MSCs, as explored by Minev *et al.* (*77*), who highlighted MSCs’ ability to carry these microcapsules while maintaining their migratory and tumor-homing abilities.

Although MSC-based drug delivery systems hold much potential, issues such as drug loading efficiency, release timing, and long-term safety must be addressed. Research by Kalimuthu *et al.* (*78*) indicates that doxorubicin-loaded MSCs retain their tumor-homing capabilities but concerns regarding sustained toxicity and immune reactions require further investigation. Moving forward, studies should aim to enhance the mechanical strength of MSC-derived carriers, optimize drug release profiles, and explore combination therapies to maximize therapeutic benefits. Despite these challenges, MSC-derived nanocarriers remain a promising approach for breast cancer treatment, offering improved targeting, increased biocompatibility, and greater overall effectiveness.

In colon cancer investigations, doxorubicin loaded on dextran-coated superparamagnetic iron oxide NPs with a crystalline iron oxide core arranged into wormlike strings was coated with bone marrow–derived MSCs. This arrangement demonstrated enhanced tumor cell uptake and, in complement binding studies, a notable (50%) decrease in C3 protein binding on membrane-coated NPs compared to uncoated ones. In addition, complement activation, as demonstrated by measurement of chemotactic agent C5a, showed an 80% reduction in release in the coated relative to uncoated NPs (*79*). Meanwhile, hollow manganese dioxide (Mn^2+^) NPs coated with human umbilical cord MSCs and functionalized with the transactivator of transcription peptide for nuclear targeting were explored for their chemoimmunotherapy potential (*80*). In this lung cancer study, the NPs delivered paclitaxel. Paclitaxel induces immunogenic cell death and, when combined with Mn^2+^, which augments cyclic guanosine 5′-monophosphate–adenosine 5′-monophosphate synthase and stimulator of interferon genes activation, created an exemplary synergetic antitumor effect (*80*). Downstream effects included dendritic cell (DC) maturation and T cell infiltration, illustrating a potent vaccine strategy.

#### Cancer cell membrane–coated NPs

Cancer cells exhibit effective immune evasion strategies including negatively charged phosphatidylserine (PS) displayed on the surface and up-regulation of CD47 and programmed death-ligand 1 (PD-L1) (*81*). They also display homotypic recognition, known as the ability of cancer cells to recognize and bind to other cells of the same type, facilitated by specific adhesion molecules on their surfaces (*82*). In lung cancer studies, PLGA NPs, loaded with paclitaxel and coated with membranes of NCI-H460 cells, effectively escaped immune recognition through CD47 present on the membranes, whereas the coating enriched with homotypic proteins (N-cadherin, galectin-3, CD44, and CD326) delivered 2.9 times more effectively toward the tumor (*83*). H1299 cell membrane–coated polymer poly(β-amino ester) core NPs carrying siRNA targeting *Polo-Like Kinase* resulted in less than 1% uptake by RAW264.7 macrophage cells, indicating an ability to evade phagocytosis and simultaneously and effectively deliver siRNA to target cells (*82*). In a cervical cancer study, siRNA/paclitaxel-coloaded PLGA NPs coated with HeLa cells were less visible to RAW264.7 cells (*84*). Low expression of proinflammatory markers (IL-1β, IL-6, and TNFα) was detected in the presence of the NPs, suggesting proficient immune evasion. In an additional cervical cancer study aimed at tackling multidrug resistance, silica NPs coated with HeLa cells delivering Ca^2+^ channel siRNA with doxorubicin reported low hemolysis, acid-responsive drug release, and decreased innate immune capture (*85*).

Collectively, cell cloaking methods show excellent promise in improving the immune acceptability of NPs for treating various diseases. However, before routine clinical successes are observed, the following aspects need to be properly addressed: (i) More detailed studies are needed to assess risks such as triggering graft versus host disease, (ii) there is insufficient research on whether cloaked NPs are protected from protein corona formation and what the implications of such formations could be, (iii) quality controls are in place to minimize batch-to-batch variability during repeat synthesis, and (iv) scalability is a realistic long-term possibility.

Notwithstanding these current challenges, two clinical trials to date are recorded on the National Clinical Trial (NTC) database—NCT02657460 and NCT01854866, respectively—both now closed (*86*). That being said, although not listed on the NTC database, Cello Therapeutics, a Biotech company in the US, is showing promise in the development of biomimetic cell membrane–coated NP, reporting that they are currently undertaking phase I clinical trials on their CE120 cancer vaccine formulation for solid cancers (*86*). They are further advertising six additional cell membrane–coated NP formulations, albeit at preclinical stage development.

#### The designer protein corona

Corona formation is a dynamic and multilayered adsorption process that occurs spatially and temporally within minutes. The van der Waals potential is said to be the driving force behind this phenomenon (*87*). Adsorbed proteins form a direct bond with the NP surface from robust electrostatic forces, maintaining stability over time. Proteins exhibiting high-affinity binding form the “hard” corona and undergo structural modifications through the development of rigid epitopes. These changes may enhance or trigger an immune response. In contrast, proteins with weak interactions form the “soft” corona continuously interact with surrounding biological fluids while maintaining their native conformation (*88*). Smaller particles are better equipped to limit corona buildup, as they have fewer atoms contributing to the van der Waals potential. Moreover, the reduced surface area helps limit protein interactions. The material of choice, and its associated Hamaker constant, also directly affects how proteins adsorb—organic materials having lower Hamaker values than metallic (*18*). Protein corona formation can be comprehensively characterized and profiled through various techniques, as outlined in Table 3.

**Table 3.** Methods used to characterize protein corona formation. CD, circular dichroism; FTIR, Fourier transform infrared spectrometry; NTA, nanoparticle tracking analysis;, TEM, transmission electron microscopy; SEM, scanning electron microscopy; GE, gel electrophoresis; FCS, fluorescence correlation spectroscopy; DCS, differential centrifugal sedimentation; DLS, dynamic light scattering; LDA, laser Doppler anemometry; TRPS, tunable resistive pulse sensing; CE, capillary electrophoresis; SERS, surface-enhanced Raman scattering; ICP-MS, inductively coupled plasma–mass spectroscopy; UV-vis, absorbance spectrometry; SPR, surface plasmon resonant; SHLS, second harmonic light scattering; FQ, fluorescence quenching; SDS-PAGE, SDS–polyacrylamide gel electrophoresis; LC-MS, liquid chromatography–mass spectroscopy, QCM, quartz crystal microbalance; SMR, suspended microchannel resonator; ITC, isothermal titration calorimetry.

Quantitative analysis method	Characteristic	Information derived from the analytical method	References
CD	Structure	The process of identifying the secondary structure of proteins	(*136*)
FTIR	Structure	Identify conformational changes in vibrational bands corresponding to amide bonds	(*136*)
NTA	Size	Using light scattering and determining changes in intensity and directional changes, larger NP movement can be tracked and recorded	(*136*)
AFM	Size	Assessment of structural element changes in the presence of mechanical forces	(*136*)
TEM and SEM	Size	Absorbed proteins can be visualized after negative staining to demonstrate morphology and size	(*136*, *137*)
GE	Size	Investigates the protein corona composition by differentiating molecular weight against a standard marker	(*136*, *138*)
FCS	Size	Measures fluctuation of fluorescence emitted from the NP, determining adsorption and dissociation coefficient of the interaction between NP and protein for colloidal morphology and concentration	(*136*, *139*)
DCS	Size and density	Measurement of sediment time through a density gradient	(*136*)
DLS	Size and surface charge	Quantify the interactions of the NP and proteins before and after binding	(*136*, *140*, *141*)
LDA	Surface charge	NP migration velocity determines the zeta potential; varying proteins and adsorption can change the zeta potential	(*136*)
TRPS	Surface charge	Maps the blockade of the electrical signal through a pore of a membrane over time, respective to protein adsorption	(*136*)
CE	Surface charge	Measures electrophoretic mobility and binding affinity of an NP in an electric field	(*136*)
SERS	Absorbance	Signal is amplified at the surface of the NP, corresponding to the Raman-active molecule (protein)	(*136*)
ICP-MS	Concentration	Calculation of the metal ions from the NP and sulfur derived from cysteine residue	(*136*)
UV-vis	Concentration (plasmon NP)	Measures the shift in wavelength and widening of the plasmon peak from protein adsorption	(*136*, *142*)
SPR	Binding affinity (nonplasmon NP)	Quantifies the binding affinity at the surface of the NP as the change in angular position of the SPR peak	(*136*, *143*, *144*)
SHLS	Binding affinity	Quantifies weakly interacting proteins through wavelength measurements; the amount of protein adsorption and the measurement of free energy are also determined	(*136*, *145*)
FQ	Fluorescence	Characterizes native fluorescence of proteins at the interface of the NP surface	(*136*, *140*, *146*)
Proteomic MS	Identification	Determine the precision number and identity of proteins adsorbed	(*136*, *139*, *143*, *144*)
SDS-PAGE	Characterization	Characterization of denatured proteins and digested disulfide bonds	(*136*, *143*)
LC-MS	Composition	Quantify the composition of proteins within the corona	(*136*, *143*)
QCM	Mass	Measures mass changes because of protein adsorption	(*136*, *140*)
SMR	Mass	Measures mass of the NP in a dynamic channel	(*136*)
ITC	Thermodynamic parameter	Measures incremental heat exchange to determine enthalpy change, binding affinity, and stoichiometry	(*136*, *147*–*149*)

Much has been reported on how protein corona formation can reduce the delivery potential of NPs. It is hard to determine the precise size of the plasma proteome; however, it is estimated that more than 10,000 proteins are included at various concentrations (*89*). Despite this, understanding of the proteins adsorbed, and how they are configured, could bring the potential to harness the process for personalized NP design. This concept of a “designer protein corona” could enhance NP development, turning a current challenge into an opportunity for more targeted and effective cancer treatments. For instance, the preadsorption of specific proteins on the NPs surface, and formation of a protein corona, has been shown to assist NPs in evading immune surveillance. However, how preformed corona NPs behave when reintroduced into an in vivo environment is less well described. This was recently explored using cationic trisodium citrate–coated gold NPs and anionic cetyltrimethylammonium bromide–coated gold NPs further coated with a primary combination of RBC and human serum albumin (HSA) and then secondary HSA coat (*89*). Both NPs were tested against controls with only a single HS coat applied. Proteomics analysis demonstrated that for the HS-only NPs, ∼72% of the proteins had functions related to immunogenic responses, while those pretreated with a combination of RBC and HS showed proteins with mostly nonimmunogenic functions. Another study has exploited the electric charge of DNA to create unPEGylated liposome/DNA complexes that encourage a proteonucleotidic corona made up of opsonin-deficient proteins to form. Human plasma concentration was found to be an important parameter in minimizing opsonins, with 5% found to be the best, keeping opsonins to less than 3% (*90*). In a contrasting approach aimed at encouraging immune reactivity, black phosphorus nanosheets were coated with serum proteins and used as immune modulators to encourage the polarization of M0 macrophages to M1 phenotypes, promoting an influx of calcium ions and inducing activations of p38 and nuclear factor κB (NF-κB) (*91*). Meanwhile, PEGylated carbon nanotubes conjugated with acute-phase plasma protein, α_1_-acid glycoprotein and immunoglobulin G producing an unfolded structure, or fibronectin for a folded structure were used to explore how the type of plasma proteins surrounding the NP affects immune response. It was found that an unfolded protein corona elevated reactive oxygen species levels and induced major proinflammatory cytokine release by macrophages. This effect stimulated innate and adaptive immune responses, particularly neutrophils, natural killer cells, and CD8^+^ T cells, showing that unfolded plasma proteins around NPs could be a way of increasing immune cell presence within the tumor microenvironment (*92*).

In conclusion, the designer protein corona holds immense potential in various cancer treatment and diagnostic fields. Once effectively harnessed, this model could be one of the most versatile and immune-friendly options, deserving of broader and more in-depth investigation.

### Sex as a biological variable in NP uptake

Sex-based differences in response to certain medications have previously been linked to genetic, anatomical, and molecular variations between females and males (*93*). This is also suggested to be true for nanotechnology applications. Despite growing recognition of the importance of considering sex as a biological variable, the impact of sex on the fate of NPs in the body is often overlooked and, hence, remains poorly understood. Nevertheless, a select few papers have begun to explore sex as a variable linked to therapeutic efficacy. Serpooshan *et al.* (*94*), for instance, demonstrated that quantum dot (QD) uptake was greater in female sex–specific human amniotic stem cells (hAMSCs) relative to male hAMSCs. Serpooshan *et al.* (*94*) hypothesized that the notable difference in QD uptake between male and female cells could be attributed to, but not limited to, (i) variations in secreted biomolecules (e.g., paracrine factors) and/or (ii) sex-related differences in cell functions and structures, such as variations in membrane composition, intracellular pathways, and cell stiffness (i.e., structural differences in the cytoskeleton). Toxicity and pharmacokinetic profiles have also shown variability between the sexes. This was elegantly demonstrated by Poley *et al.* (*93*) in their 2022 review. In addition, NP clearance rates have also been shown to differ between male and female rodents, with females showing slower clearance rates for liposomes loaded with topoisomerase-I inhibitor (CKD602) or topotecan (*95*). In a separate study, this was also demonstrated for liposomal doxorubicin (*96*). Time of administration during the reproductive cycle has also shown to give vastly different biodistribution patterns during NP treatment in rodents. In one of the few studies of its kind, Poley *et al.* described that what stage NP treatment was administered during the reproductive cycle was critical to therapeutic efficacy in breast cancer. Specifically, during the estrus stage, NPs preferentially accumulated at the ovaries of mice rather than at the breast tumor site. In light of such findings, studies of this kind should be more frequently observed in literature given the efficacy implications for cancers external to the ovaries. Moreover, more in-depth studies should be performed to assess whether NP-delivered ovarian cancer treatment should be more attuned toward delivery during a specific phase of the menstrual cycle.

Substantial sex-specific differences have also been observed in respect of protein corona profiles, specifically, presence or absence of proteins and other analyses involved in metabolic activity (*97*). In a proof-of-concept silica NP study that looked at protein corona profiles in men and women shows that protein corona formation is influenced by the size and porosity of the structure in both male and female protein coronas. Increasing porosity leads to a higher number of unique proteins, likely due to the greater active surface area of mesoporous 100 NPs compared to Stöber NPs, which provides more surfaces for protein adsorption (*98*). Nearly all of the identified sex-specific proteins in the corona profiles, regardless of the size and porosity of the silica NPs used, were more prevalent in male coronas, suggesting that the diagnostic potential of these silica NPs may be greater for males than for females. Studies of this kind for other types of NP compositions are sparse in the literature; however, these could provide valuable information in relation to translational potential and dosing strategies and thus should be considered.

Last, sex may dramatically affect how innate and adaptive immune cells respond in relation to NP composition and charge as demonstrated by Vulpis *et al.* (*99*) using Dioleoyl-3-trimethylammonium propane (DOTAP) LNPs. They observed that uptake of NPs by natural killer and lymphocyte cells was greater in male cells. However, when the immune cells were subsequently pretreated with human plasma, these effects were diminished, suggesting that such sex-based differences could be managed through presoaking with a standardized blend of human plasma. Given DOTAP is highly cationic, future studies should attempt to evaluate the effect in mixed LNPs and polymeric NPs to determine how and whether charge and composition are influential in male immune cell uptake.

### Incorporating mechanobiology for future cancer nanomedicines

While the approaches discussed above have advanced our understanding of NP–immune system interactions, there remain notable challenges in achieving optimal therapeutic outcomes. A key limitation has been the insufficient consideration of biomechanical factors that influence NP behavior in physiological environments. Mechanobiology—the study of how forces and mechanical cues influence biological processes—offers valuable insights into these complex nano-bio interactions. By understanding the mechanical microenvironment and associated biochemical signaling pathways, we can better interpret how cells sense and respond to NPs beyond traditional physicochemical characterizations. Integrating mechanobiological principles into NP design therefore represents a promising frontier for enhancing cancer therapeutic delivery, efficacy, and specificity.

#### Advanced biomechanical nanotools for probing nano-bio interfaces

**Fig. 6.**
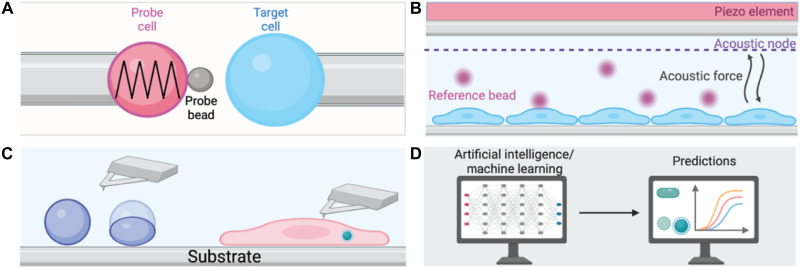
Biomechanical nanotools and analysis to further improve NP characterizations and understand bio-nano interactions. (**A**) Graphic illustrating a biomembrane force probe (BFP), used to determine dissociation kinetics. (**B**) Acoustic force spectroscopy, a way to quantify the strength of cell–extracellular matrix adhesion. (**C**) Atomic force microscopy (AFM), topographical mapping, and characterization of the NPs at high resolution. (**D**) Artificial intelligence, machine learning, and computational fluid dynamics (CFD) modeling, enhancing understanding of and predicting NP behavior in vivo.

The use of sophisticated biomechanical nanotools unlocks potential in studying nano-cell interactions before and after protein corona formation, which is crucial for accurate understanding of the functionality of third-generation nanomaterials (Fig. 6).

One of the greatest tools in this instance is biomembrane force probes (BFPs). First developed by Evan Evans in the mid-1990s (*100*), BFPs have undergone a progressive evolution, culminating in the emergence of ultrastable BFPs able to dissect the slow dissociation kinetics occurring between a therapeutic and its target, offering a valuable quantitative measure of clinical efficacy (*101*). Similarly, acoustic force spectroscopy, a technique used to produce acoustic waves that register force to microbeads tethered to a surface, presents a way to quantify the strength of cell–extracellular matrix adhesion (*102*). Effectively interpreting these interactions is integral to understanding how nanomaterials can navigate and influence the tumor microenvironment.

Atomic force microscopy (AFM) is acknowledged as a valuable tool in NP characterizations. This method enables precise topographical mapping and characterization of the NPs at high resolution, offering more than 1000 times the resolution of optical microscopy and generating three-dimensional images of sample surfaces. Scanning of NPs is done through a cantilever, whereby the interaction force occurring between the cantilever tip and the NPs surface is measured to generate an image and determine the Young’s modulus—the property that determines NPs’ stiffness. AFM also provides important information about specific cell mechanics issues, including interactions with cell membranes and the internalization dynamic of NPs (*103*).

Computational approaches are increasingly vital for NP development. Computational fluid dynamics (CFD) modeling uses algorithms approximating fluid dynamics, such as the Navier-Stokes equations (*104*), to optimize NP characteristics such as size, shape, surface charge, and material composition. Artificial intelligence and machine learning are also driving important previously unknown understandings of NP behavior in vivo through elaborate predictive algorithms. The integration of these computational tools with experimental approaches is arguably one of the most exciting frontiers in nanomedicine development.

#### Mechanical design principles for enhanced biological performance

For many years, controlling basic physicochemical properties such as size, shape, and charge was the primary approach to influence NP biological behavior. However, mechanobiology has revealed the importance of more nuanced physical attributes such as stiffness, flexibility, and surface texture in determining biological interactions (*105*). For example, soft NPs are said to interact less with immune cells and are not easily destroyed or removed by macrophages (*106*).

A recent study explored the impact of stiffness for silica nanocapsules endowed with a bone marrow–derived MSC coat (*107*). The two different silica nanocapsules displayed Young’s moduli of 44 MPa and 2.3 GPa, respectively. Softer membrane coatings provided better immune evasion, as evidenced by reduced macrophage uptake. Soft-membrane NPs also had better formed MSC coats than their stiffer counterparts and higher CD90 and CXCR4 receptor presence. These attributes were considered to be a result of the deformation of the soft silica nanocapsules during the mesoporous calcium silicate nanoparticles (MCSN) fabrication process. Compared to stiffer membrane-coated nanocapsules, the deformed shape also improved cancer cell uptake via the receptor-mediated pathway. Last, protein corona formation varied between stiff and soft coatings, with more rigid coatings attracting a greater amount of complement protein (C3) and immunoglobulin proteins. However, a study that explored silica NPs ranging from 560 kPa to 1.18 GPa (*108*) reports opposite findings. Here, silica nanocapsules were modified with methoxy-PEG. A targeting group was then further functionalized with folate (FA)–PEG. Surface density of FA-PEG–modified silica nanocapsules was around 0.09 molecules/nm^2^. Those with higher Young’s moduli were internalized more quickly; however, the effect of NP elasticity on cellular uptake was dependent on the type of cell-NP interaction. In macrophage phagocytosis and receptor-mediated endocytosis, the elasticity of NPs played a crucial role in their uptake by cells. However, cancer cells became less sensitive to stiffness during nonspecific clathrin- and caveolin-independent endocytosis.

Natural delivery systems such as viruses, blood cells, and pollen exhibit anisotropy, meaning they have nonuniform tailored structures with direction-dependent properties that enhance their functional capabilities in biological processes. Nanoparticle design has begun to embrace this idea for greater targeted delivery with an ever-increasing complexity. For example, asymmetric organic/inorganic nanohybrids composed of soft organic polystyrene-*b*-poly(4-vinylpyridine)-*b*-poly(ethylene oxide) single micelle nanosphere (12 nm in size and 632 MPa in Young’ modulus) and stiff inorganic silicon dioxide nanobulge (∼8 nm and 2275 MPa) (*109*) were recently tested for uptake efficiency in four T1 breast cancer cells. Uptake was markedly higher (~3 times) in the asymmetric NPs compared to their symmetrical equal. These results were further echoed in additional cancer cell lines (HeLa, 786-O, and CT26). The number of protruding nanorods was found to be important for uptake, with a single protrusion deemed most effective over multiple nanorods. Uptake for asymmetric NPs was also increased in macrophage cell line RAW264.7, relative to the symmetrical NPs, suggesting that they may be a model delivery system suited toward reprogramming of TAMs. TAMs are known to provide an anti-inflammatory environment, which helps tumors to develop, metastasize, and become chemoresistant. Reprogramming toward a proinflammatory phenotype promises to be a highly effective way of targeting tumors. The NP delivery field is playing a leading role in this immune system–focused approach, and mechanobiology is central to this, answering important questions, most notably how and whether nanomechanics can be modeled to trigger specific cell behaviors. A report on how macrophages alter their biological function in response to the elasticity of silica NPs found that although macrophages showed a preference for phagocytosis of stiffer NPs, in vivo, softer silica NPs were better suited to penetrating tumors and reprogramming TAMs. The softer NPs induced less membrane deformation than the stiffer and activated mechanosensitive ion channel protein Piezo1, leading to calcium influx and activation of the NF-κB pathway known to modulate several proinflammatory genes (*110*). This seminal study presents as one of the first to showcase how specific NP mechanical properties can be harnessed to trigger an intratumoral proinflammatory response against cancer cells.

#### Surface roughness and its multifaceted influence on NP performance

Quantifying surface roughness at the nanoscale is essential across manufacturing, engineering, and scientific disciplines due to its substantial impact on physical and chemical interactions. Surface topography can markedly influence how materials interact with light, phonons, molecules, and biological systems, thereby altering their functional properties and performance.

In NP-based delivery systems, surface roughness and porosity are critical considerations. Rough surfaces have been shown to reduce interparticle cohesion and contact area, thereby improving dispersibility—an important advantage for inhalation formulations, where efficient aerosolization is key. For targeting tumors, rough surfaces have been thought of as favorable because they create strong nano-tumor interactions. This was demonstrated by Xue *et al.* (*111*), who used hydrofluoric acid etching on silica-based core-shell NPs carrying bromodomain-containing protein 4 inhibitor, JQ-1. In a melanoma model (C57BL/6 mice), the adhesive properties of polydopamine enabled tumor antigens to bind to the NP surface, facilitating their uptake by DCs. These DCs subsequently migrated to lymph nodes to activate cytotoxic T lymphocytes (CTLs). Simultaneously, JQ-1@PSNs-R suppressed PD-L1 expression, alleviating tumor-associated immunosuppression and enabling the CTLs to target and destroy remaining tumor cells.

Supporting this concept, Grundler *et al.* (*112*) demonstrated that modifying surface roughness using bottlebrush block copolymers much improved circulation time and tumor accumulation via enhanced extravasation and tissue distribution, as compared to smooth-surfaced analogs. Conversely, Kim *et al.* (*113*) reported that in human lung adenocarcinoma epithelial cells (A549), layered double hydroxide NPs with smooth surfaces exhibited more effective cellular uptake than their rough counterparts at a concentration of 200 μg/ml.

Other studies suggest that NP surface morphology must be tuned on the basis of the delivery context. For instance, hybrid organic-inorganic NPs with small, spherical, and smooth surfaces were found to better evade immune detection and were generally regarded as more biocompatible (*114*). Surface roughness also affects protein corona formation, influencing both the quantity and viscoelastic properties of adsorbed proteins—factors shaped by protein type, concentration, and binding affinity (*115*). These early protein-NP interactions can exacerbate immune responses and hinder NP transport across critical physiological barriers before reaching target tissues such as tumors.

Together, these findings underscore the importance of tailoring surface roughness to the intended biological application. In response to this need, viruslike nanocarriers with dynamically variable surface roughness have recently emerged as promising platforms, offering tunable interfaces to optimize each stage of the delivery pathway.

Bagheri *et al.* (*116*) present an excellent example of this kind of approach. Under normal physiological conditions, the rough, spiked surface of the mesoporous silica shell NPs doped with lanthanide is hidden inside of 16 polymer layers formed by positively charged poly (diallyldimethyl-ammonium chloride and negatively charged poly(allylamine)-dimethylmaleic anhydride, which self assembles through electrostatic interaction. To provide greater biocompatibility, a final layer composed of polyethyleneglycol-poly(allylamine)-dimethylmaleic anhydride with an additional PEG segment was added. The polymer camouflage generated a temporary smooth surface, which helped evade nonspecific interactions; however, once the NPs reach the acidic tumor microenvironment, the polymer camouflage was uncoupled from the nanocarrier to expose the rough surface (spikes), simultaneously switching the surface charge of the nanocarrier from negative to positive, encouraging strong nano-tumor interactions and delivering photodynamic therapy (PDT). The results of this study demonstrate a lengthened circulatory time and 100% greater tumor accumulation relative to the control nanocarrier with a permanent rough surface. Surface-roughness–trans-formable nanocarriers present as an excellent approach to greater tumor delivery as long as the detailed synthesis can be made fully reproducible, cost effective and readily upscalable.

Spiked NPs have demonstrated potential; however, precisely how spike formation dominates cell interactions is still somewhat of an enigma. In attempts to fill this knowledge gap, a recent study looked at two NPs with different length spikes (7 and 12 nm, respectively) and compared them against rough-surfaced spherical NPs. The spiky core of NPs was developed using a “DNA-engineered seeded growth” approach (*117*), with the polyadenylate tail single-stranded DNA–modified NPs (15 nm) used as seeds. Surfactant (polyvinylpyrrolidone), reductant (hydroxylamine hydrochloride), and precursor gold(III) chloride permitted the growth of spikes with tunable spikiness on their surface. Overall, the spiky formation led to rapid uptake through myosin IIA recruitment, resulting in a 4.6-fold increase in cellular uptake efficiency and a switch to clathrin-independent endocytosis. In addition, trafficking speed was greatly increased. Spike length was a determining factor, as the NPs with the longest spikes being most rapidly endocytosed.

Collectively, tailoring surface roughness, either static or dynamic, is key to improving NP performance for targeted therapy, immune evasion, and efficient delivery across biological barriers now and in the future.

#### Engineering for complex physiological flow conditions

Understanding fluid mechanics is crucial yet challenging for successful NP development. Intravenously delivered particles experience varying shear stress conditions (0.5 to 30 dynes/cm^2^) and complex flow patterns that influence their distribution, protein corona formation, and ultimate therapeutic efficacy (*118*). The parabolic velocity flow speed within the vessels is highest at the center and decreases toward the walls, and these conditions directly influence protein corona formation (*18*). Following permeation through the walls, NPs then need to overcome interstitial flow in the extracellular matrix to reach target cells. Collectively, the dynamic interplay in the blood and extracellular matrix can change the NPs identity, affecting its immunogenicity, ability to target specific sites, and therapeutic outcome.

One study has investigated this effect using docetaxel-loaded gelatin-oleic NPs—developed to target cancer cells through bioorthogonal click chemistry under physiological biomimetic conditions. The NPs were developed using 1-ethyl-3-(3-dimethylaminopropyl) carbodiimide and *N*-hydroxysuccinimide covalent chemistry, followed by loading of docetaxel via the incubation method and inclusion of coumarin-6 for imaging. Within the biomimetic microfluidic chamber where breast cancer cells (MCF7) were attached, the medium containing the NPs flowed through for 45 min under laminar flow. The cells experienced shear stress of 5 dynes/cm^2^. Under these physiologically relevant test conditions, NPs’ efficacy was noticeably reduced (*118*). When an HSA-bound protein corona was added to the NPs surface, efficacy was reduced even further relative to a static model. In a lung cancer study, a fattigation (conjugating fatty acids to other molecules) platform developed from a gelatin–oleic acid conjugate loaded with coumarin-6, and paclitaxel was examined for uptake in A549 cells under different levels of shear stress (0.5, 5, and 50 dynes/cm^2^) in the biomimetic microfluidic system (*119*).

In contrast to the previous study, under such physiological flow conditions, the NPs’ drug delivery was improved over the static condition. The highest shear stress condition, 50 dynes/cm^2^, showed the greatest improvement in efficacy. While there remain some issues surrounding reproducibility and accuracy of in vivo stress conditions (*120*), integrating this technology early on in the design process could create more realistic NPs’ translational contenders that are more adept at navigating the complex and dynamic biological environment.

As it stands, the causal relationship between quantifiable mechanical properties of NPs, such as Young’s modulus, and their biological effects is not yet established. While studies suggest that mechanical properties can influence cellular interactions, uptake, and toxicity (*121*), definitive evidence demonstrating a direct cause-and-effect relationship remains limited. Further research is needed to fully elucidate how specific mechanical characteristics directly affect biological responses in various contexts.

### Final thoughts

Over the past 5 years, the global impact of nanomedicines has been immeasurable; however, there remain fundamental issues that cloud successes and hinder clinical translation of future game-changer therapies, particularly those targeted toward intravenous delivery. While universally safeguarding against toxicity and manufacturing, scalability and storage remain ongoing areas of development; NPs have failed in clinical trials not only because of formulation issues but also because of inadequate patient stratification and superficial clinical trial design. The concept of companion diagnostics is increasingly recognized as a key strategy to address these challenges. This is elegantly described by Gawne *et al.* (*122*), who discuss the prospects of imaging modalities, positron emission tomography, and magnetic resonance imaging as methods to determine whether NPs preferentially accumulate in tumors before therapeutic administration, thereby aiding in the identification of patients who are likely to respond and derive genuine benefit from nanomedicine. This approach is especially pertinent when the EPR effect is anticipated to play a leading role. Many early clinical trials have fallen short due to the absence of such stratification strategies, resulting in inconsistent outcomes and an underestimation of nanomedicine’s true potential. Incorporating this perspective would offer a more comprehensive view of the translational barriers faced by NP-based therapies.

Other notable aspects moving forward should include filling the gaps in knowledge that exist, and effective reporting of these findings should take precedence. Influential papers arising in the last year come from Joyce *et al.* (*123*) and Caputo *et al.* (*124*), who both call for a better framework describing the core principles and critical biological hurdles present through at the lack of harmonized analytical methods and reference materials. This issue was emphasized during a high-level workshop in Paris in October 2023, which brought together experts from metrology institutes, regulatory agencies, industry, and academia. Participants agreed on the importance of a unified approach to standardization, involving organizations such as International Organization for Standardization, European Committee for Standardization (CEN), American Society for Testing and Materials, Versailles Area Materials and Standards, and regulators. The focus is on high-priority nanomedicines, particularly LNPs and liposomal formulations used in mRNA vaccines and drug delivery, as well as metal oxide NPs for diagnostics and radiotherapy. The outcome was a proposal to establish a dedicated working group within CEN/TC 352 to coordinate European standardization efforts, ensuring that they are aligned with global regulatory expectations and facilitating the broader acceptance of nanomedicines. Meanwhile, in the US, the Nanotechnology Characterization Laboratory founded in 2004 has created a comprehensive standardized analytical framework that encompasses physicochemical characterization along with preclinical evaluation of the immunological, pharmacological, and toxicological properties of NPs and devices. The analytical assay cascade is freely available to researchers globally (*125*).

**Fig. 7.**
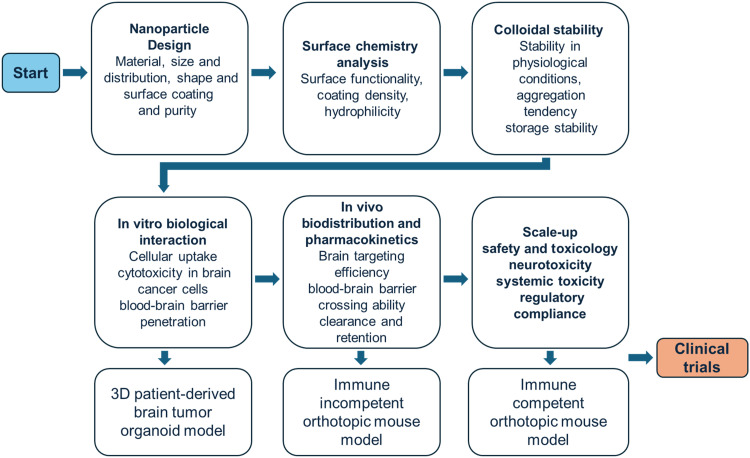
Flowchart illustrating a comprehensive standardized framework for the development of an NP-delivered therapeutic targeting brain cancer. The framework encompasses key stages including NP design (e.g., material, size, shape, and surface functionality), preclinical evaluation (in vitro and in vivo testing for blood-brain barrier penetration, biodistribution, and efficacy), clinical translation considerations (scale-up, safety, and regulatory compliance), and feedback-driven optimization for improved therapeutic outcomes.

Furthermore, to align with the growing emphasis on human-relevant testing methods, the integration of advanced in vitro systems such as organoids and organ-on-a-chip models, combined with artificial intelligence–driven analytics, is poised to play a pivotal role in NP evaluation. This direction is strongly supported by the FDA’s recent announcement in April 2025, outlining a strategic plan to replace animal testing for monoclonal antibody therapies and other drugs with more predictive and ethical alternatives. These cutting-edge platforms offer unprecedented physiological relevance and scalability, making them ideally suited for assessing the safety, efficacy, and biodistribution of complex nanomedicine formulations. As the field moves toward harmonization and standardization, embedding such technologies within regulatory frameworks will not only accelerate development timelines but also enhance translational fidelity in NP-based drug delivery. An example of how this may look for a study aimed toward brain cancers is shown in Fig. 7.

Looking forward, the integration of artificial intelligence and advanced computational modeling with experimental approaches will likely accelerate the development of more effective nanomedicines. The growing understanding of mechanobiological principles, combined with immune-conscious design, promises to yield NPs capable of better navigating biological barriers while maintaining their therapeutic functionality. Success in this endeavor will require continued collaboration across disciplines, from materials science and engineering to immunology and clinical medicine. As we move forward, focusing on both immune and mechanical aspects of nano-bio interactions will be crucial for developing the next generation of cancer nanomedicines that can effectively translate from bench to bedside.
